# Exploration of the Prognostic Markers of Multiple Myeloma Based on Cuproptosis‐Related Genes

**DOI:** 10.1002/cnr2.70151

**Published:** 2025-03-05

**Authors:** Xiao‐Han Gao, Jun Yuan, Xiao‐Xia Zhang, Rui‐Cang Wang, Jie Yang, Yan Li, Jie Li

**Affiliations:** ^1^ Department of Hematology Hebei General Hospital Shijiazhuang China

**Keywords:** cuproptosis, hematology, immunity, multiple myeloma, prognosis

## Abstract

**Background:**

The investigation of cuproptosis in relation to tumor development has been limited, particularly in multiple myeloma (MM), indicating the need for further research. Our study aimed to examine the impact of cuproptosis‐related genes (CRGs) on the prognosis of MM.

**Methods:**

Using the datasets, we filtered cuproptosis score‐related differentially expressed genes (CRDEGs) by overlapping the DEGs between the MM and normal groups and between the high and low cuproptosis score groups. Additionally, key module genes were identified through weighted gene co‐expression network analysis. A univariate Cox algorithm and multivariate Cox analysis were employed to obtain biomarkers of MM and build a prognostic model before conducting independent prognostic analysis.

**Results:**

A total of 59 CRDEGs were filtered, demonstrating their involvement in the COPII vesicle coat and endoplasmic reticulum protein processing, and protein processing in the endoplasmic reticulum. Six prognosis‐related biomarkers (PARP1, EDEM3, SEC23A, RSL24D1, TTC37, and SRP72) were obtained, and a prognostic model was developed. The performance of the model was verified using a test cohort (GSE136324 dataset) and a validation cohort (GSE24080 dataset). Risk score, age, albumin, International Staging System (ISS) score, and β2‐microglobulin (B2M) were found to be significant predictors of prognosis independently.

**Conclusion:**

As a result of this investigation, a set of six biomarkers associated with cuproptosis (PARP1, EDEM3, SEC23A, RSL24D1, TTC37, and SRP72) were screened to provide a basis for predicting the prognosis of MM.

## 1 Introduction

Multiple myeloma (MM) presents as a type of blood cancer, representing 10% of all hematological malignancies [1] and causing more than 10 000 deaths annually [2]. Characterized by the abnormal secretion of immunoglobulins and expansion of clonal plasma cells in the bone marrow (BM), MM presents with clinical features such as hypercalcemia, renal impairment, anemia, and bone lesions [3]. Recent advancements in medical treatments, such as the increasing usage of proteasome inhibitors, immunomodulators, and monoclonal antibodies, have significantly enhanced the survival rates of individuals with MM [4, 5]. However, there has been no significant progress in assessing prognostic indicators in patients with MM. Furthermore, the response to treatment is variable given the large heterogeneity of MM. A more accurate prognosis assessment will help to specify individualized diagnosis and treatment measures. Therefore, it is essential to explore new biomarkers to distinguish patients with MM with different prognoses and identify novel therapeutic targets.

Efforts to devise treatment plans that target cancer cell destruction through the initiation of cellular demise are a prominent area of research within the realm of clinical oncology. Previous studies have shown that cancer cell apoptosis resistance is the main reason for the failure of tumor therapy, and it is more effective to eliminate cancer cells through the non‐apoptotic regulatory cell death (RCD) pathway than through the apoptotic RCD pathway [6]. A vital constituent for bodily metabolism, copper acts as a crucial factor in the operation of numerous enzymes within the body. Copper can trigger macroautophagy or autophagy, and it is a lysosome‐dependent degradation pathway that plays a dual role in regulating the survival or death of cells under various stress conditions. Additionally, the involvement of copper contributes to the process of programmed cell death. Moreover, cell death is induced when the number of copper ions in the cells is too high. Tsvetkov et al. [7] recently proposed that copper‐induced cell death, termed cuproptosis, targets fatty acylated tricarboxylic acid (TCA) cycle proteins and ultimately inhibits the respiratory regulatory function of mitochondria to induce cell death [7, 8]. Cuproptosis is a distinct form of cell death, differing from cell death caused by other mechanisms such as apoptosis, ferroptosis, and necrosis. The main process of cuproptosis depends on the level of copper ions in the cell. When excess Cu^2+^ enters the cell, it is transported to the mitochondria and reduced to Cu^+^. This Cu^+^ interferes with the TCA and electron transport chain, leading to oligomerization of fatty acylated proteins and loss of FeS cluster proteins, ultimately leading to cell death. Research has identified a notable variance in the copper levels found in the bloodstream of individuals diagnosed with tumors as opposed to those who are considered healthy [9, 10, 11]. Copper can promote the growth and metastasis of tumors and is closely related to the occurrence and development of cancer [12, 13]. Studies have confirmed the value of copper chelators as therapeutic drugs that breakthrough drug‐resistance bottlenecks in breast cancer, liver cancer, lung cancer, and melanoma [14]. Cuproptosis inducers can trigger the process of cuproptosis in cells. For example, illisto uses copper ions as a carrier to induce cuproptosis in cells by introducing copper ions into cells and interfering with FeS cluster biosynthesis, which has a potential anti‐cancer effect. cuproptosis provides a new perspective for tumor treatment. However, the mechanism of cuproptosis in MM remains a puzzle.

Utilizing the MM transcriptome and clinical data from the Gene Expression Omnibus (GEO) database, a series of biomarkers related to cuproptosis in MM were identified. A deeper understanding of the molecular mechanisms of cuproptosis in MM will help the subsequent development of new molecular targeted drugs. It can provide new treatment ideas for MM, especially for relapsed and refractory MM, and help to improve the efficacy of MM.

## 2 Materials and Methods

### 2.1 Data Collection for Patients With MM

Datasets such as GSE47552 [15], GSE136324 [16], and GSE24080 [17, 18, 19] were extracted from the GEO database. Utilizing the GSE47552 dataset (GPL6244), which consisted of the RNA‐seq data of BM from five normal individuals and 44 MM cohorts, was aimed at conducting a comparative analysis. Concurrently, the GSE136324 dataset (GPL27143) was employed for survival analysis, encompassing the RNA‐seq data of whole bone marrow (WBM) sourced from 867 MM samples. Moreover, data from the GSE24080 dataset (GPL570), which consisted of RNA‐seq data from BM plasma cells of 558 MM samples, served as an independent validation set. Subsequently, 10 cuproptosis‐related genes (CRGs) were obtained from a previous report [7].

### 2.2 Identifying Key Module Genes by Employing WGCNA Filtering Techniques

The cuproptosis score was computed for each sample in the GSE136324 dataset via the ssGSEA algorithm, and all samples were classified into high and low cuproptosis score groups. Meanwhile, the analysis of overall survival was carried out by correlating with the patients' survival data in MM. The co‐expression network was constructed using WGCNA (v 1.69) [20] based on the GSE136324 dataset.

### 2.3 Exploring and Identifying Differentially Expressed Genes Related to the Cuproptosis Score Through Screening and Enriching Their Functional Relevance

Initially, the limma package (v 3.44.3) was utilized to identify differentially expressed genes (DEGs) between the MM and normal groups [21] In the GSE47552 dataset according to *p* < 0.05 and |log_2_FC| > 0.5. Meanwhile, DEGs between the high and low cuproptosis score groups were selected in the GSE136324 dataset with *p* < 0.05 and |log_2_FC| > 0.5. The findings from the contrast analysis are visually presented using a volcano plot. The visualization showcases the expression patterns of the top 100 DEGs. The cuproptosis score‐related differentially expressed genes (CRDEGs) were screened by overlapping the DEGs between the MM and normal groups with those between the high and low cuproptosis score groups, in addition to key module genes. Enrichment analyses of CRDEGs using the clusterProfiler package(v 3.16.0) were conducted for gene ontology (GO) and Kyoto Encyclopedia of Genes and Genomes [22].

### 2.4 Construction and Assessment of the Prognostic Model

The samples of the GSE136324 dataset were classified into training and test cohorts at a ratio of 7:3 (training cohort = 607, test cohort = 259). The univariate Cox algorithm [23] was used for CRDEGs to acquire candidate genes, which were then subjected to multivariate Cox analysis. The genes gained were used as biomarkers in this study. Patients were classified into high‐ and low‐risk groups according to the optimal truncation values of the risk score computed from the cuproptosis‐related biomarkers: Riskscore = $\sum_{1}^{n}\text{coef}\left(\text{genes}\right)*\text{expression}\;\left(\text{genes}\right)$. Kaplan–Meier (K–M) survival curves were drawn, and the survival ROC package (v 1.16.2) [24] was utilized to compute the area under the curve (AUC) values for receiver operating characteristic (ROC) curves to assess the predictive accuracy of the model. The correlation between risk score and clinical characteristics was assessed using the chi‐square test. The statistics were illustrated by a heatmap. The prognostic model was verified using a test cohort and an external validation cohort (GSE24080 dataset).

### 2.5 Independent Prognostic Analysis

Primary analysis was conducted on clinicopathological variables and the prognostic model within a subset of 607 cases from the GSE136324 dataset's training group. Following this, a nomogram was developed and visually represented, with an assessment of the model's performance conducted through calibration curve analysis.

### 2.6 Screening and Gene Set Enrichment Analysis (GSEA) of Risk‐Related DEGs

Risk‐related DEGs were selected in the training cohort of the GSE136324 dataset with *p* < 0.05 and |log_2_FC| > 0.5. Subsequently, GSEA was performed to identify the enriched regulatory pathways and biological functions of each DEG with |NES| > 1, NOM *p* < 0.05, and *q* < 0.25. Finally, the top 10 results for GO and KEGG significance were visualized separately.

### 2.7 Immuno‐Microenvironmental Analysis

Utilizing the CIBERSORT algorithm (v 1.03), the analysis was performed to calculate the percentage of 22 different immune cell subtypes in each sample. This calculation was performed within the training cohort of the GSE136324 dataset. A visualization representing the associations among different types of immune cells was generated. Following this, a comparison was made between the differential immune cells found in both groups, with a box plot being generated. Following this, an analysis was conducted to assess the relationship between biomarkers and the different immune cells utilizing the Spearman method, with the outcomes being visually represented.

### 2.8 Statistical Analysis

The analysis of all biological information was conducted using the R programming language. Utilizing the Wilcoxon test, comparisons were made among the data from various groups.

## 3 Results

### 3.1 Grouping of High and Low Cuproptosis Scores and Screening of Key Module Genes

**FIGURE 1:**
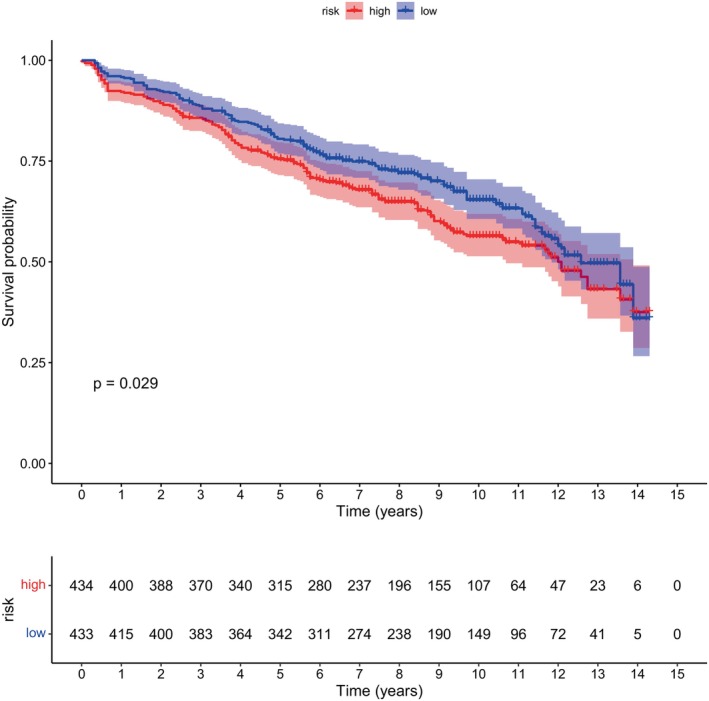
Survival with high and low copper mortality scores. According to the median cuproptosis score, the GSE136324 samples were divided into a high‐risk group and a low‐risk group, and the overall survival time between the two groups was significantly different. The blue curve represents the low‐risk group, the red curve represents the high‐risk group, and there was a significant difference in survival time between the two groups (*p* = 0.029, *p* < 0.05).

Each sample in the GSE136324 dataset was classified into high‐ and low‐score groups (high‐score group, *n* = 434, low‐score group, *n* = 433) according to the median cuproptosis scores computed by the ssGSEA algorithm. A more favorable outlook was observed among those with lower cuproptosis scores (Figure 1).

### 3.2 Screening and Functional Enrichment of CRDEGs

**FIGURE 2:**
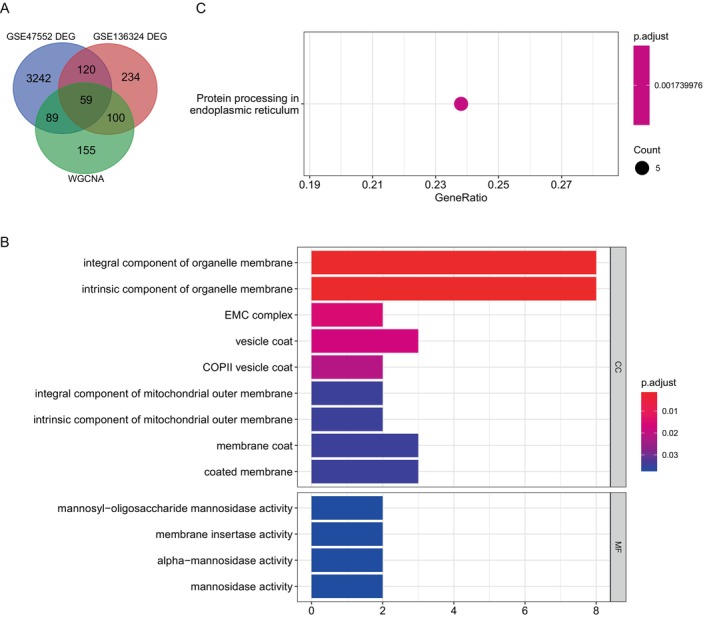
The pathways involved in differential genes were explored through enrichment analysis. The differentially expressed cuproptosis genes may primarily play a role in protein folding and processing in the endoplasmic reticulum. (A) The intersection of three gene sets (GSE47552, GSE136324, and WGCNA) yielded 59 genes, all of which were differentially expressed cuproptosis genes. (B) Enrichment analysis obtained 13 significant GO items (*p* < 0.05). (C) Enrichment analysis of the 59 genes revealed one significant Kyoto Encyclopedia of Genes and Genomes pathway, namely, protein processing in the endoplasmic reticulum (*p* = 0.00173).

A total of 3510 DEGs were identified between the MM and normal groups, along with 513 DEGs between the high and low cuproptosis score groups. A total of 59 CRDEGs, including key module genes, were identified through the overlap of DEGs from the MM and normal groups, as well as from the high and low cuproptosis score groups (Figure 2A).

The potential functions of the 59 CRDEGs were predicted using GO and KEGG pathway analysis. The GO term annotation indicated that NRDEGs primarily participated in the COPII vesicle coat, EMC complex, vesicle coat, and integral component of organelle membrane (Figure 2B). KEGG enrichment results included protein processing in the endoplasmic reticulum (Figure 2C).

### 3.3 Biomarker Screening and Prediction Model

**FIGURE 3:**
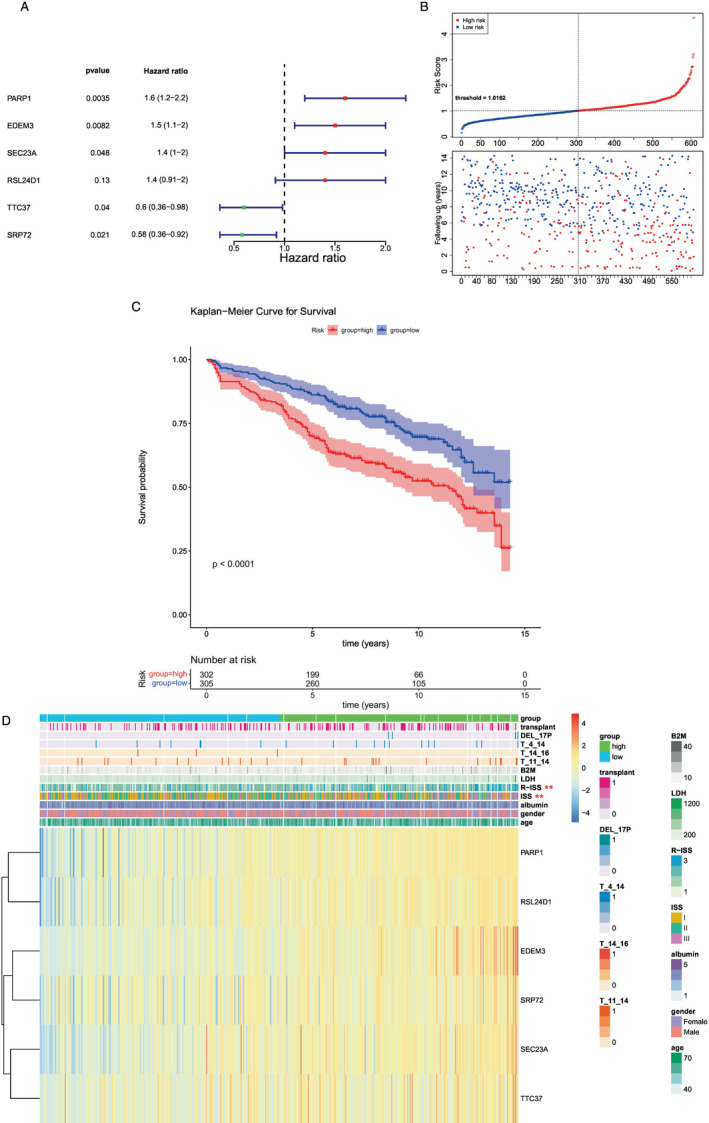
Cox regression analysis. (A) After multivariate Cox analysis, a total of six genes were found to be significant. (B) Cuproptosis risk increased progressively from left to right. Samples were divided according to the median value into high‐ and low‐risk groups. Blue represents the low‐risk group, while red represents the high‐risk group; the low‐risk group had a longer follow‐up. (C) Red represents the high‐risk group and blue represents the low‐risk group. Survival analysis of the high‐ and low‐risk groups is depicted in the following figure, which showed that there were significant differences in survival (*p* = 0.035, *p* < 0.05). (D) Clinical traits are listed at the top of the heatmap, with the first line illustrating the risk group (blue for low risk, green for high risk). Each small square in the heatmap represents a gene, with color intensity indicating gene expression levels (darker colors indicate higher expression levels).

In total, six prognosis‐related biomarkers (PARP1, EDEM3, SEC23A, RSL24D1, TTC37, and SRP72) were acquired by univariate and multifactor Cox analyses (Figure 3A). Patients were classified into high‐ and low‐risk groups on the basis of the optimal truncation values (training cohort = 1.016153; test cohort = 1.177333; external validation cohort = 1.129844). Additionally, survival data of the patients were analyzed, and risk curves were generated (Figure 3B). Survival analysis curves showed that the low‐risk group of the training cohort had a higher survival rate than the high‐risk group (Figure 3C). The results of the ROC curves revealed that the model had a decent predictive performance with AUCs > 0.6 (1‐, 3‐, and 5‐years). By comparing the different proportions of the two groups of patients in the different subgroups, it was found that ISS and R‐ISS were significant (Figure 3D).

**FIGURE 4:**
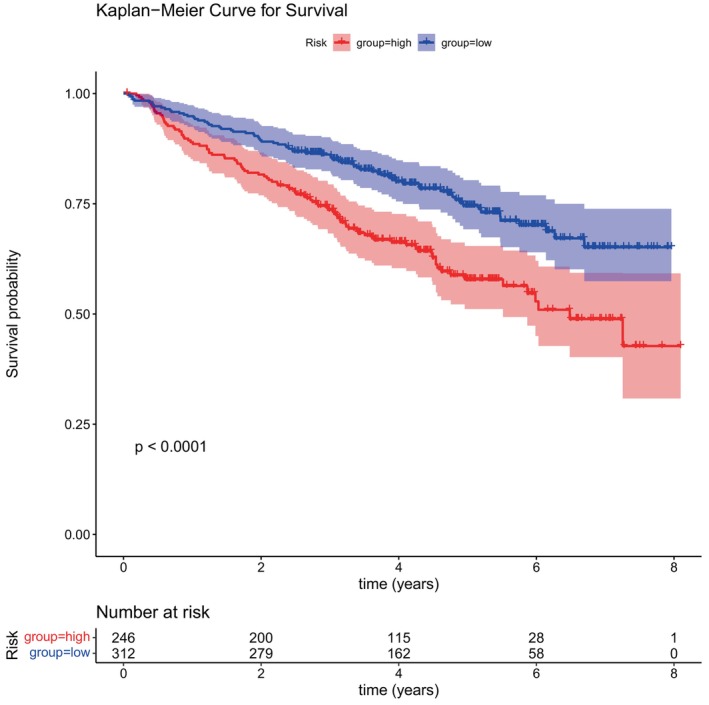
K‐M survival curve. According to the expression levels of six genes, the risk score for each patient was calculated, and patients were subsequently divided into two groups according to the risk scores. Red represents the high‐risk group, and blue represents the low‐risk group. Survival analysis comparing the high‐ and low‐risk groups revealed significant differences in terms of survival (*p* < 0.05).

Subsequently, the model's predictive performance was assessed using a test cohort and an external validation cohort. Demonstrated by the risk profile plots and survival curves in the test cohort, findings were in line with those from the training cohort (Figure 4). The AUC values for the test cohort were all > 0.6, with similar results observed for the external validation cohort.

### 3.4 Identification of Independent Prognostic Factors and Model Evaluation

**FIGURE 5:**
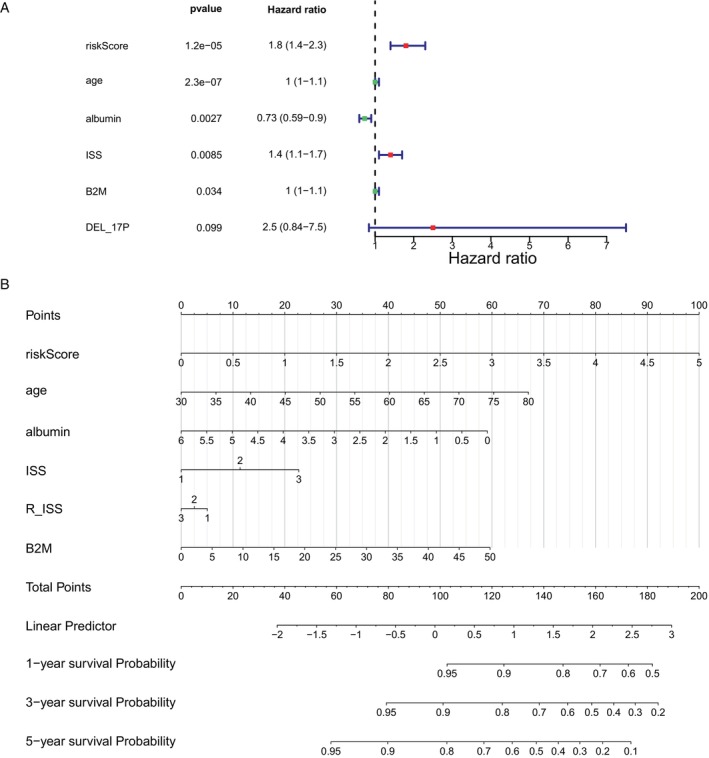
Risk model of independent prognosis. (A) Through multifactor Cox analysis, we identified 5 factors that showed a significant correlation with survival (risk score, age, albumin, ISS, and B2M). (B) Each factor is assigned a score, and the total score is the sum of all scores from all factors. Based on the total score, estimated 1‐year, 3‐year, and 5‐year survival rates are calculated. A higher total score indicates a lower predicted survival rate.

In total, five significant factors (risk score, age, albumin, ISS, and B2M) were identified after analytical screening (Figure 5A). Utilizing the quintet of significant predictors, a predictive nomogram was employed to estimate patient OS over the course of 1, 3, and 5 years (Figure 5B). As illustrated by the calibration curve, the precision of the nomogram was decent and optimally predicted at 1 year.

### 3.5 GSEA of Risk‐Related DEGs

**FIGURE 6:**
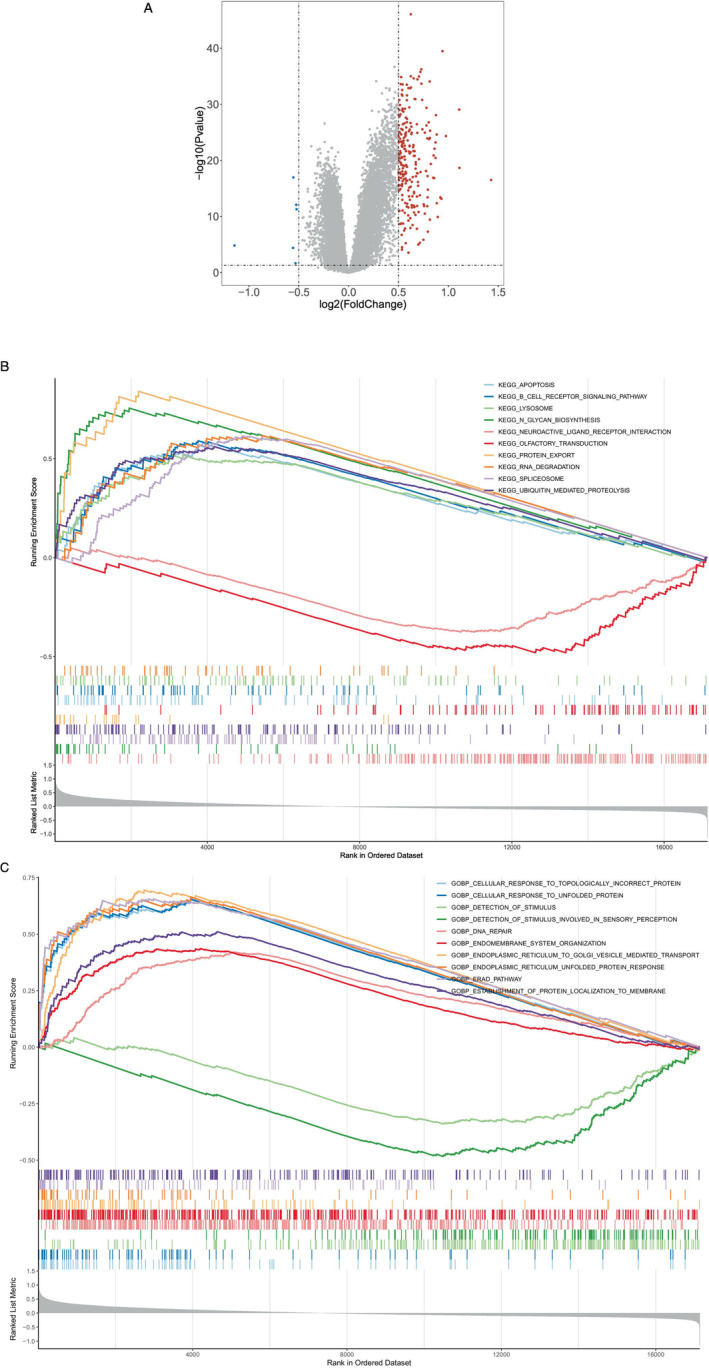
Differential and enrichment analyses of high‐ and low‐risk groups. (A) Volcano plot comparing the gene expression between the high and low sample groups. (B, C) The high and low expression groups exhibited enrichment in the immune‐related KEGG and GO pathways, respectively.

A total of 239 significantly differentially expressed genes were identified by enrichment analysis in the comparison between high‐ and low‐risk groups (Figure 6A). GSEA was performed to explore the access regulatory pathways and molecular functions of risk‐related DEGs. Significant pathways identified in the KEGG enrichment analysis included regulation of cell death, signaling cascade mediated by B cell receptor, and organelle degradation processes (Figure 6B). DEGs were mainly enriched in GO terms such as cellular response to topologically incorrect protein, detection of stimulus, and cellular response to unfolded protein (Figure 6C).

### 3.6 Immune Infiltration Analysis

**FIGURE 7:**
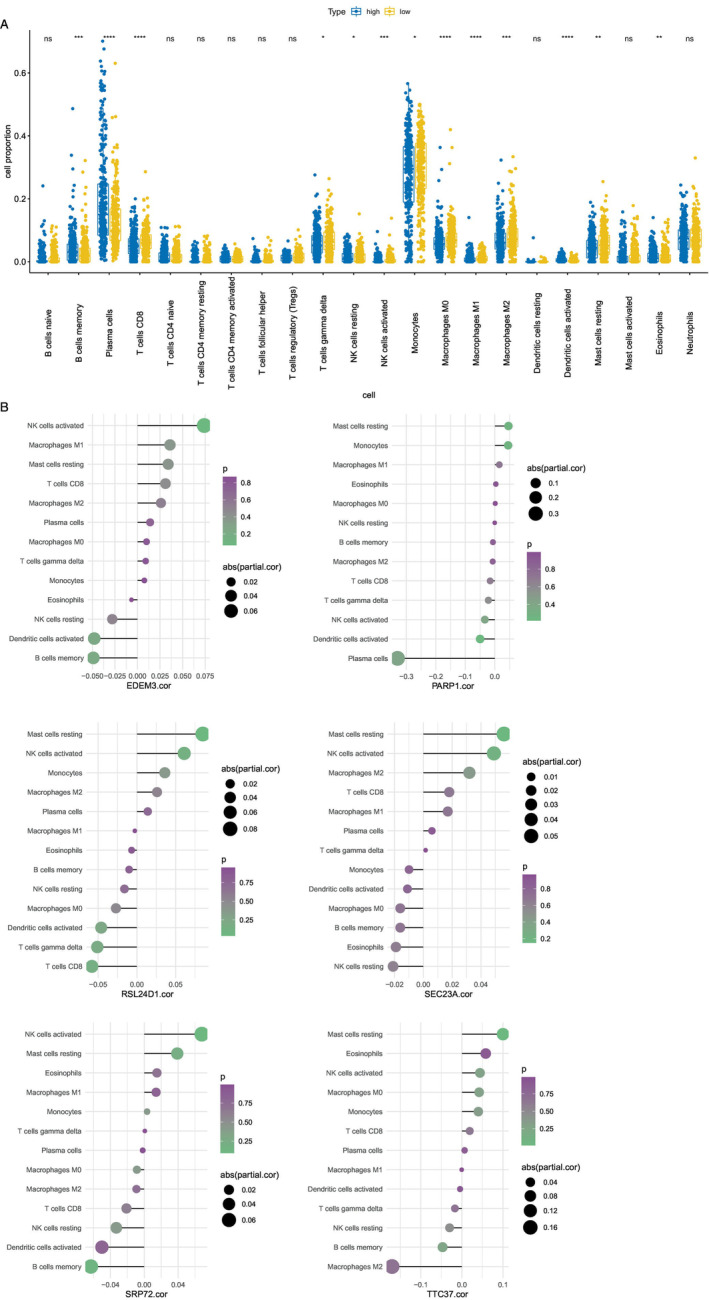
CIBERSORT algorithm employed to infer the abundance of immune cells. (A) A total of 13 of the 22 types of immune cells exhibited significant variances, including B cell memory, plasma cells, CD8 T cells, gamma delta T cells, resting NK cells, activated NK cells, monocytes, M0 macrophages, M1 macrophages, M2 macrophages, activated dendritic cells, resting mast cells, and eosinophils. (B) The correlation between these 13 significant immune cell types and the six prognostic genes was calculated and mapped separately.

The representation of the 22 immune cell types in each sample is illustrated by the bars, while the heatmap depicts the relationships among these immune cells. The box plot indicated 13 types of immune‐infiltrating cells (Figure 7A). The analysis of correlations unveiled that EDEM3 and SRP72 demonstrated positive associations with activated NK cells and displayed negative associations with memory B cells (Figure 7B), while PARP1 was negatively correlated with plasma cells (Figure 7B). Furthermore, RSL24D1 was positively associated with resting mast cells and negatively associated with CD8 T cells (Figure 7B). SEC23A was positively associated with resting mast cells (Figure 7B), and TTC37 was negatively correlated with M2 macrophages (Figure 7B).

## 4 Discussion

MM is a malignant hematologic tumor that originates from B cells and is second only to lymphoma in terms of incidence. In 2020, the number of new MM cases and deaths globally reached 176 404 and 117 077 [6], respectively, and will continue to rise. Abnormal proliferation of plasma cells is the primary cause of MM, which leads to the inhibition of BM hematopoietic function, resulting in clinical manifestations such as anemia, renal dysfunction, hypercalcemia, and bone destruction. Although the clinical application of proteasome inhibitors, immunomodulators, and CD38 monoclonal antibodies has prolonged the OS and PFS of patients, the disease's complexity and interaction with the BM microenvironment contribute to high rates of relapse among patients. thus, the challenge of finding a definitive cure for MM persists [25]. Therefore, searching for new prognostic markers and exploring therapeutic targets are current research hotspots.

Matuszczak [26] suggests that essential elements play a crucial role in anti‐cancer protective mechanisms. Zinc and copper have been recognized as influential in the occurrence of cancer, such as breast and ovarian cancer. Within the cell, the presence of zinc ions influences the functioning and stability of the p53 protein. When zinc levels are too high, they disrupt the structure of the p53 protein and reduce its ability to bind to DNA. Copper has the ability to outcompete zinc for its typical binding position on p53, which can cause improper protein folding and interfere with the normal function of p53 [27]. Copper participates in antioxidant pathways that protect lipids from peroxidation. It helps maintain the balance between oxidized and reduced forms of molecules within cells. In cancer cells, the level of copper manifests in two distinct biological traits: stimulating tumor development by promoting cell proliferation, metastasis, and angiogenesis, and it can also induce programmed cell death in tumor cells, thereby impeding tumor progression.

Based on the clinical traits and genomic data of patients with MM in the GEO database, we identified six CRGs (*PARP1*, *EDEM3*, *SEC23A*, *RSL24D1*, *TTC37*, and *SRP72*) that were significantly associated with prognosis. The correlation regression coefficient was used to calculate the risk score, and a further risk model was constructed to better predict the prognosis of MM survival. Within our investigation, the main focus of our investigation was on the enrichment of the six CRGs in cellular processes related to protein handling within the endoplasmic reticulum, involving the structural framework of the COPII vesicle coat, playing a crucial role in the structure of the mitochondrial outer membrane, Bag lamination, along with the presence of alpha–mannosidase functionality.

PARP1 belongs to the family of poly (ADP‐ribose) polymerase enzymes, which are primarily involved in the DNA damage repair process. PARP Super Series plays a crucial role in the repair mechanism of DNA damage. Through the activation of the nuclear factor NFAT, it has the capability to influence the differentiation of CD4+ T cells. Additionally, in collaboration with Foxp3, it modulates the TGF‐β receptor expression in CD4+ T cells, leading to the activation of its poly ADP‐ribosylation process. *PARP1* protects macrophages from oxidation‐induced death. PARP inhibitors have been reported to achieve good results in treating ovarian cancer, gastric cancer, and other solid tumors [28]. High cytotoxic concentrations of copper have been associated with the induction of oxidative DNA damage and the impairment of repair mechanisms for oxidative DNA damage caused by visible light at lower noncytotoxic levels. Copper can induce DNA strand breaks, where poly (ADP‐ribosyl)ation is the first nuclear event following DNA strand break induction. *PARP1* is believed to mediate the main part of poly (ADP‐ribosyl)ation. It has already been reported that the activity of isolated *PARP1* is strongly inhibited by copper [29]. Combined with the above research results, it is speculated that the overexpression of *PARP1* may lead to the uncontrolled proliferation of MM tumor cells, thus leading to the occurrence of MM [30, 31, 32].

The relationship between EDEM3, SEC23A, RSL24D1, TTC37, SRP72, and copper has not been studied previously. Involved in the processing of misfolded glycoproteins, EDEM3 functions as a mannosidase that removes mannose residues, targeting them for degradation by the ERAD pathway. Furthermore, the upregulation of certain genes related to the response of improperly folded proteins has been noted in prostate cancer [33, 34, 35]. It has been reported that high concentrations of copper trigger an unfolded protein response, resulting in increased protein damage, decreased oxidative phosphorylation, and enhanced ROS, leading to mitochondrial damage [36] and ultimately cell death. The region of 14q21 is where SEC23A is positioned. Consisting of 22 exons, this gene plays a role in building COPII and facilitating the transfer of the majority of secreted proteins from the endoplasmic reticulum matrix to the Golgi apparatus. The function of SEC23A extends to playing a role in the pathogenesis and progression of various types of solid tumors [37, 38, 39]. The level of SEC23A has been shown to be inversely correlated with follicular helper T cells, Tregs, activated NK cells, and myeloid dendritic cells [40]. SEC23A inhibits the self‐renewal of melanoma CSCs by inactivating ER phagocytosis. Mechanistically, inhibition of SEC23A reduces endoplasmic reticulum stress, thereby reducing FAM134B‐induced endoplasmic reticulum phagocytosis. Cancer stem cells can use the SEC23A/ER stress/FAM134B/ER phagocytosis axis for self‐renewal. Therefore, if the expression level of SEC23A can be inhibited, it can block tumor stem cell renewal [41].

Initially recognized for its role in the biogenesis process of the 60S ribosomal subunit, RSL24D1 was first identified. Depletion of RSL24D1 results in the disruption of pre‐ribosomal RNA (pre‐rRNA) transcription, causing a reduction in protein synthesis and stabilization of p53 in human cells [42]. TTC37 mutation is primarily studied in hairy hepatic enteric syndrome, and there has been no evidence of an association with tumorigenesis [43]. SRP72 is part of the signal recognition particle (SRP) complex, responsible for targeting proteins in the endoplasmic reticulum. It has been reported that SRP72 is involved in the binding of SRP receptors and the bearing of signal sequences for newly translated proteins into the lumen of the endoplasmic reticulum [44]. When SRP72 cannot bind to SRP68, it may lead to incorrect translocation of proteins destined for the cell membrane or extracellular space, leading to tumorigenesis. The discovery of the primary hydrophobic, concave bonding region on SRP72 linked to SRP68 opens doors for the creation of compounds that could be utilized in treating cancer. Overall, all six crucial genes were found to be involved in the development and progression of tumors through protein processing in the endoplasmic reticulum.

In this study, six genes that may be associated with the prognosis of MM were screened through dataset analysis. By drawing the ROC curve, we confirmed the validity of the risk model (AUC > 0.6), and then verified it using an external dataset, indicating that the novel risk model could effectively predict the prognosis of MM. As expected, the enrichment analysis of GO and KEGG pathways was significantly associated with DNA repair, the ERAD pathway, and the endoplasmic reticulum unfolded protein response. These pathways are closely related to tumorigenesis. Our findings provide a new direction for the treatment and prognosis of MM. However, the above‐mentioned genes have been rarely studied in hematological tumors, and further experimental mechanism research is needed to obtain further confirmation and to strive to provide new ideas for the treatment of MM. The prognosis of MM is related to many factors, including age, the general condition of patients, the number of myeloma cells, the development stage of the disease, and the response to treatment, etc. Although the outcome of MM has been greatly improved with the current research on new drugs, almost all patients are facing relapse, and the prognosis of MM patients still needs to be improved. Appropriate targets should be selected for the precise treatment of MM. The prognosis model proposed in this study is intended to supplement the existing prognosis models.

## Author Contributions

Conceptualization: Xiao‐Han Gao and Jie Li. Formal analysis: Xiao‐Han Gao and Jie Li. Data curation: Xiao‐Han Gao. Software: Xiao‐Han Gao and Jun Yuan. Resources: Xiao‐Xia Zhang, Yan Li, and Jie Li. Visualization: Jie Yang, Yan Li, and Jie Li. Project administration: Rui‐Cang Wang. Writing – original draft: All authors. Writing – review and editing: Xiao‐Han Gao and Jie Li.

## Disclosure

All authors have completed the ICMJE uniform disclosure form.

## Ethics Statement

The data of this study were obtained from open databases without ethical committee approval.

## Conflicts of Interest

The authors declare no conflicts of interest.

## Supporting information


Data S1.

## Data Availability

The data that support the findings of this study are openly available in “GEO” at https://www.ncbi.nlm.nih.gov/geo/query, reference number [15, 16, 17, 18, 19].

## References

[1] T. B. Olesen , I. T. Andersen , A. G. Ording , et al., “Use of Bisphosphonates in Multiple Myeloma Patients in Denmark, 2005–2015,” Support Care Cancer 29 (2021): 4501–4511, 10.1007/s00520-020-05934-8. 33458807

[2] C. Varga , M. Maglio , I. M. Ghobrial , et al., “Current Use of Monoclonal Antibodies in the Treatment of Multiple Myeloma,” Brit J HAEMATOL 181 (2018): 447–459, 10.1111/bjh.15121. 29696629

[3] C. H. Cuffe , M. B. Quirke , and C. McCabe , “Patients' Experiences of Living With Multiple Myeloma,” British Journal of Nursing 29, no. 2 (2020): 103–110, 10.12968/bjon.2020.29.2.103. 31972106

[4] K. C. Anderson , “Vision Statement for Multiple Myeloma: Future Directions,” Cancer Treatment and Research 169 (2016): 15–22, 10.1007/978-3-319-40320-5_2. 27696255

[5] P. Neri , N. J. Bahlis , C. Paba‐Prada , et al., “Treatment of Relapsed/Refractory Multiple Myeloma,” Cancer Treatment and Research 69 (2016): 169–194, 10.1007/978-3-319-40320-5_10. 27696263

[6] J. Huang , S. C. Chan , V. Lok , et al., “The Epidemiological Landscape of Multiple Myeloma: A Global Cancer Registry Estimate of Disease Burden, Risk Factors, and Temporal Trends,” Lancet Haematol 9 (2022): 670–677, 10.1016/S2352-3026(22)00165-X. 35843248

[7] P. Tsvetkov , S. Coy , B. Petrova , et al., “Copper Induces Cell Death by Targeting Lipoylated TCA Cycle Proteins,” Science 375, no. 6586 (2022): 1254–1261. 35298263 10.1126/science.abf0529 PMC9273333

[8] X. Ren , C. Pan , Z. Pan , et al., “Knowledge Mapping of Copper‐Induced Cell Death: A Bibliometric Study From 2012 to 2022,” Medicine 101 (2022): 31133, 10.1097/MD.0000000000031133. PMC10662818 36397452

[9] S. Tsymbal , A. Refeld , V. Zatsepin , and O. Kuchur , “The p53 Protein Is a Suppressor of Atox1 Copper Chaperon in Tumor Cells Under Genotoxic Effects,” PLoS One 18 (2023): 0295944, 10.1371/journal.pone.0295944. PMC10735018 38127999

[10] H. Huang , Z. Lv , L. Yang , et al., “Development and Validation of Cuproptosis Molecular Subtype‐Related Signature for Predicting Immune Prognostic Characterization in Gliomas,” J Cancer res CLIN 149 (2023): 11499–11515, 10.1007/s00432-023-05021-5. PMC11796944 37392200

[11] Z. Wang , D. Jin , S. Zhou , et al., “Regulatory Roles of Copper Metabolism and Cuproptosis in Human Cancers,” Frontiers in Oncology 13 (2023): 1123420, 10.3389/fonc.2023.1123420. 37035162 PMC10076572

[12] Y. Bengtsson , K. Demircan , J. Vallon‐Christersson , et al., “Serum Copper, Zinc and Copper/Zinc Ratio in Relation to Survival After Breast Cancer Diagnosis: A Prospective Multicenter Cohort Study,” Redox Biology 63 (2023): 102728, 10.1016/j.redox.2023.102728. 37210781 PMC10209876

[13] T. L. Turan , H. J. Klein , J. Hackler , et al., “Serum Selenium‐Binding Protein 1 (SELENBP1) in Burn Injury: A Potential Biomarker of Disease Severity and Clinical Course,” Antioxidants (Basel) 12 (2023): 1927, 10.3390/antiox12111927. 38001780 PMC10669776

[14] Y. Liu , X. Guan , M. Wang , et al., “Disulfiram/Copper Induces Antitumor Activity Against Gastric Cancer via the ROS/MAPK and NPL4 Pathways,” Bioengineered 13, no. 3 (2022): 6579–6589. 35290151 10.1080/21655979.2022.2038434 PMC9278967

[15] L. López‐Corral , L. A. Corchete , M. E. Sarasquete , et al., “Transcriptome Analysis Reveals Molecular Profiles Associated With Evolving Steps of Monoclonal Gammopathies,” Haematologica 99, no. 8 (2014): 1365–1372, 10.3324/haematol.2013.087809. 24816239 PMC4116836

[16] S. A. Danziger , M. McConnell , J. Gockley , et al., “Bone Marrow Microenvironments That Contribute to Patient Outcomes in Newly Diagnosed Multiple Myeloma: A Cohort Study of Patients in the Total Therapy Clinical Trials,” PLoS Medicine 17, no. 11 (2020): e1003323, 10.1371/journal.pmed.1003323 Erratum in, *PLoS Medicine*, 2021;18(9):1003774. 33147277 PMC7641353

[17] V. Popovici , W. Chen , B. G. Gallas , et al., “Effect of Training‐Sample Size and Classification Difficulty on the Accuracy of Genomic Predictors,” Breast Cancer Research 12, no. 1 (2010): R5, 10.1186/bcr2468. 20064235 PMC2880423

[18] L. Shi , G. Campbell , W. D. Jones , et al., “The MicroArray Quality Control (MAQC)‐II Study of Common Practices for the Development and Validation of Microarray‐Based Predictive Models,” Nature Biotechnology 28, no. 8 (2010): 827–838, 10.1038/nbt.1665. PMC3315840 20676074

[19] J. S. Mitchell , N. Li , N. Weinhold , et al., “Genome‐Wide Association Study Identifies Multiple Susceptibility Loci for Multiple Myeloma,” Nature Communications 1, no. 7 (2016): 12050, 10.1038/ncomms12050. PMC4932178 27363682

[20] P. Langfelder and S. Horvath , “WGCNA: An R Package for Weighted Correlation Network Analysis,” BMC Bioinformatics 29, no. 9 (2008): 559. 10.1186/1471-2105-9-559 PMC2631488 19114008

[21] M. Ritchie , B. Phipson , W. Di , et al., “Limma Powers Differential Expression Analyses for RNA‐Sequencing and Microarray Studies,” Nucleic Acids Research 43, no. 7 (2015): e47. 25605792 10.1093/nar/gkv007 PMC4402510

[22] T. Wu , E. Hu , S. Xu , et al., “Clusterprofiler 4.0: A Universal Enrichment Tool for Interpreting Omics Data,” Innovation (N Y) 2, no. 3 (2021): 100141. 10.1016/j.xinn.2021.100141 PMC8454663 34557778

[23] Y. Ye , Q. Dai , and H. Qi , “A Novel Defined Pyroptosis‐Related Gene Signature for Predicting the Prognosis of Ovarian Cancer,” Cell Death Discovery 7, no. 1 (2021): 71. 33828074 10.1038/s41420-021-00451-x PMC8026591

[24] X. Robin , N. Turck , A. Hainard , et al., “pROC: An Open‐Source Package for R and S+ to Analyze and Compare ROC Curves,” BMC Bioinformatics 12 (2011): 77. 21414208 10.1186/1471-2105-12-77 PMC3068975

[25] C. Varga , J. P. Laubach , K. C. Anderson , et al., “Investigational Agents in Immunotherapy: A New Horizon for the Treatment of Multiple Myeloma,” British Journal of Haematology 181 (2018): 433–446, 10.1111/bjh.15116. 29748955

[26] M. Matuszczak , A. Kiljańczyk , W. Marciniak , et al., “Antioxidant Properties of Zinc and Copper‐Blood Zinc‐To Copper‐Ratio as a Marker of Cancer Risk BRCA1 Mutation Carriers,” Antioxidants (Basel) 13, no. 7 (2024): 841, 10.3390/antiox13070841. 39061909 PMC11273827

[27] A. Formigari , E. Gregianin , and P. Irato , “The Effect of Zinc and the Role of p53 in Copper‐Induced Cellular Stress Responses,” Journal of Applied Toxicology 33 (2013): 2854, 10.1002/jat.2854. 23401182

[28] A. Rizvi and I. Naseem , “Causing DNA Damage and Stopping DNA Repair ‐ Vitamin D Supplementation With Poly(ADP‐Ribose) Polymerase 1 (PARP1) Inhibitors May Cause Selective Cell Death of Cancer Cells: A Novel Therapeutic Paradigm Utilizing Elevated Copper Levels Within the Tumour,” Medical Hypotheses 144 (2020): 110278, 10.1016/j.mehy.2020.110278. 33254582

[29] T. Schwerdtle , I. Hamann , G. Jahnke , et al., “Impact of Copper on the Induction and Repair of Oxidative DNA Damage, Poly(ADP‐Ribosyl)ation and PARP‐1 Activity,” Molecular Nutrition & Food Research 51 (2007): 201–210, 10.1002/mnfr.200600107. 17230584

[30] J. van der Veeken , A. Glasner , Y. Zhong , et al., “The Transcription Factor Foxp3 Shapes Regulatory T Cell Identity by Tuning the Activity of Trans‐Acting Intermediaries,” Immunity 53, no. 5 (2020): 971–984, 10.1016/j.immuni.2020.10.010. 33176163 PMC8363055

[31] T. Rakitina , A. Zeifman , F. Novikov , et al., “Novel PARP1 Inhibitors Potentiate Doxorubicin Antitumor Activity In Vitro,” Mendeleev Communications 25 (2023): 364–366, 10.1016/j.mencom.2015.09.016.

[32] Y. H. Huang , S. J. Yin , Y. Y. Gong , et al., “PARP1 as a Prognostic Biomarker for Human Cancers: A Meta‐Analysis,” Biomarkers in Medicine 15 (2021): 1563–1578, 10.2217/bmm-2020-0891. 34651514

[33] E. Scott , R. Garnham , K. Cheung , A. Duxfield , D. J. Elliott , and J. Munkley , “Pro‐Survival Factor EDEM3 Confers Therapy Resistance in Prostate Cancer,” International Journal of Molecular Sciences 23, no. 15 (2022): 8184. 35897761 10.3390/ijms23158184 PMC9332126

[34] K. Hirao , Y. Natsuka , T. Tamura , et al., “EDEM3, a Soluble EDEM Homolog, Enhances Glycoprotein Endoplasmic Reticulum‐Associated Degradation and Mannose Trimming,” Journal of Biological Chemistry 281 (2006): 1200, 10.1074/jbc.M512191200. 16431915

[35] J. Zhang , B. Zheng , X. Zhou , et al., “Increased BST‐2 Expression by HBV Infection Promotes HBV‐Associated HCC Tumorigenesis,” Journal of Gastrointestinal Oncology 12 (2021): 694–710, 10.21037/jgo-20-356. 34012659 PMC8107608

[36] H. Huo , S. Wang , Y. Bai , et al., “Copper Exposure Induces Mitochondrial Dynamic Disorder and Oxidative Stress via Mitochondrial Unfolded Protein Response in Pig Fundic Gland,” Ecotoxicology and Environmental Safety 223 (2021): 112587, 10.1016/j.ecoenv.2021.112587. 34352579

[37] J. Ing , B. Wang , and P. Liu , “The Functional Role of SEC23 in Vesicle Transportation, Autophagy and Cancer,” International Journal of Biological Sciences 15, no. 11 (2019): 2419–2426. 31595159 10.7150/ijbs.37008 PMC6775307

[38] Q. Cheng , K. Liu , J. Xiao , et al., “SEC23A Confers ER Stress Resistance in Gastric Cancer by Forming the ER Stress‐SEC23A‐Autophagy Negative Feedback Loop,” Journal of Experimental & Clinical Cancer Research 42 (2023): 232, 10.1186/s13046-023-02807-w. 37670384 PMC10478313

[39] C. Li , L. Zhao , Y. Chen , et al., “Microrna‐21 Promotes Proliferation, Migration, and Invasion of Colorectal Cancer, and Tumor Growth Associated With Down‐Regulation of Sec23a Expression,” BMC Cancer 16 (2016): 605, 10.1186/s12885-016-2628-z. 27495250 PMC4974737

[40] S. Zhaoran , C. S. Linnebacher , and M. Linnebacher , “Increased SEC23A Expression Correlates With Poor Prognosis and Immune Infiltration in Stomach Adenocarcinoma,” Cancers (Basel) 15, no. 7 (2023): 2065. 37046730 10.3390/cancers15072065 PMC10093042

[41] Z. Sun , D. Liu , B. Zeng , et al., “Sec23a Inhibits the Self‐Renewal of Melanoma Cancer Stem Cells via Inactivation of ER‐Phagy,” Cell Communication and Signaling: CCS 20, no. 1 (2022): 22. 35236368 10.1186/s12964-022-00827-1 PMC8889648

[42] M. A. McCool , A. F. Buhagiar , C. J. Bryant , et al., “Human Pre‐60S Assembly Factors Link rRNA Transcription to Pre‐rRNA Processing,” RNA 29 (2022): 82–96, 10.1261/rna.079149.122. 36323459 PMC9808572

[43] A. Fabre , C. Martinez‐Vinson , B. Roquelaure , et al., “Novel Mutations in TTC37 Associated With Tricho‐Hepato‐Enteric Syndrome,” Human Mutation 32 (2011): 277–281, 10.1002/humu.21420. 21120949

[44] Y. Gao , Q. Zhang , Y. Lang , et al., “Human Apo‐SRP72 and SRP68/72 Complex Structures Reveal the Molecular Basis of Protein Translocation,” Journal of Molecular Cell Biology 9, no. 3 (2017): 220–230. 28369529 10.1093/jmcb/mjx010 PMC5907831

