# Supplementary material for: Exploration of the Prognostic Markers of Multiple Myeloma Based on Cuproptosis‐Related Genes
Source: Cancer Rep (Hoboken). 2025 Mar 5;8(3):e70151. doi: 10.1002/cnr2.70151 (PMC11880913; doi:10.1002/cnr2.70151)
Supplement: Supplementary file 1 — Data S1. [file CNR2-8-e70151-s001.docx]

Software list.

| Software | Version |
| --- | --- |
| Limma | 3.44.3 |
| Ggplot2 | 3.3.2 |
| Pheatmap | 0.7.7 |
| WGCNA | 1.69 |
| ClusterProfiler | 3.16.0 |
| Survival | 3.2–3 |
| pROC | 1.16.2 |
| rms | 5.1–4 |
| CIBERSORT | 1.03 |
